# Analysis and application of a suite of recombinant endo-β(1,3)-d-glucanases for studying fungal cell walls

**DOI:** 10.1186/s12934-021-01616-0

**Published:** 2021-07-03

**Authors:** Vanessa S. D. Carvalho, Laura Gómez-Delgado, M. Ángeles Curto, M. Belén Moreno, Pilar Pérez, Juan Carlos Ribas, Juan Carlos G. Cortés

**Affiliations:** Instituto de Biología Funcional y Genómica Zacarías González, 2. CSIC and Universidad de Salamanca, 37007 Salamanca, Spain

**Keywords:** Recombinant endo-β(1,3)-d-glucanase, β(1,3)-d-glucan, Cell wall, Fission yeast, Fungi

## Abstract

**Background:**

The fungal cell wall is an essential and robust external structure that protects the cell from the environment. It is mainly composed of polysaccharides with different functions, some of which are necessary for cell integrity. Thus, the process of fractionation and analysis of cell wall polysaccharides is useful for studying the function and relevance of each polysaccharide, as well as for developing a variety of practical and commercial applications. This method can be used to study the mechanisms that regulate cell morphogenesis and integrity, giving rise to information that could be applied in the design of new antifungal drugs. Nonetheless, for this method to be reliable, the availability of trustworthy commercial recombinant cell wall degrading enzymes with non-contaminating activities is vital.

**Results:**

Here we examined the efficiency and reproducibility of 12 recombinant endo-β(1,3)-d-glucanases for specifically degrading the cell wall β(1,3)-d-glucan by using a fast and reliable protocol of fractionation and analysis of the fission yeast cell wall. This protocol combines enzymatic and chemical degradation to fractionate the cell wall into the four main polymers: galactomannoproteins, α-glucan, β(1,3)-d-glucan and β(1,6)-d-glucan. We found that the GH16 endo-β(1,3)-d-glucanase PfLam16A from *Pyrococcus furiosus* was able to completely and reproducibly degrade β(1,3)-d-glucan without causing the release of other polymers. The cell wall degradation caused by PfLam16A was similar to that of Quantazyme, a recombinant endo-β(1,3)-d-glucanase no longer commercially available. Moreover, other recombinant β(1,3)-d-glucanases caused either incomplete or excessive degradation, suggesting deficient access to the substrate or release of other polysaccharides.

**Conclusions:**

The discovery of a reliable and efficient recombinant endo-β(1,3)-d-glucanase, capable of replacing the previously mentioned enzyme, will be useful for carrying out studies requiring the digestion of the fungal cell wall β(1,3)-d-glucan. This new commercial endo-β(1,3)-d-glucanase will allow the study of the cell wall composition under different conditions, along the cell cycle, in response to environmental changes or in cell wall mutants. Furthermore, this enzyme will also be greatly valuable for other practical and commercial applications such as genome research, chromosomes extraction, cell transformation, protoplast formation, cell fusion, cell disruption, industrial processes and studies of new antifungals that specifically target cell wall synthesis.

**Supplementary Information:**

The online version contains supplementary material available at 10.1186/s12934-021-01616-0.

## Introduction

Fungal cells are surrounded by a thick and rigid structure, namely the cell wall. The function of this structure is to protect against environmental changes, such as osmotic or temperature stress, that can cause cell alterations. However, despite its robustness, the cell wall is an extremely dynamic structure with a great plasticity and phenotypic diversity [1–3]. The cell wall composition directly affects its function; therefore, it is fundamentally important to identify the components involved and determine their contribution to this structure. In addition, the architecture and composition of the cell wall is specific for each fungal species, and the composition can change depending on the site of the cell wall, during the cell cycle or in response to environmental disturbances [2, 3].

The fungal cell wall is predominantly composed by polysaccharides [4, 5], and there are different methods based on either chemical or enzymatic degradation that can be used to analyze its composition. The main examples for chemical degradation are alkali solubilization [6, 7], acid hydrolysis [8, 9], periodate oxidation [10], borohydride reduction [6, 11], Smith degradation [12], permethylation [9] and carboxymethylation [13]. However, these methods are more laborious to carry out than those based on enzymatic degradation and only provide information about the type of bonds between the monosaccharides forming the polymers, but not about the amount and type of polymers forming the cell wall [14]. On the other hand, the use of carbohydrate-degrading enzymes greatly facilitates the analysis and quantification of cell wall polysaccharides [15]. Both methods are complimentary, and as such the combination of chemical and enzymatic analyses together with the usefulness of radioactive labeled cell walls is the most comprehensive method for quantifying the different cell wall polymers [16]. Additionally, other strategies that have provided valuable information on the composition and construction of the cell wall generally focus on the amount and type of bonds between monosaccharides [9, 17, 18].

**Fig. 1:**
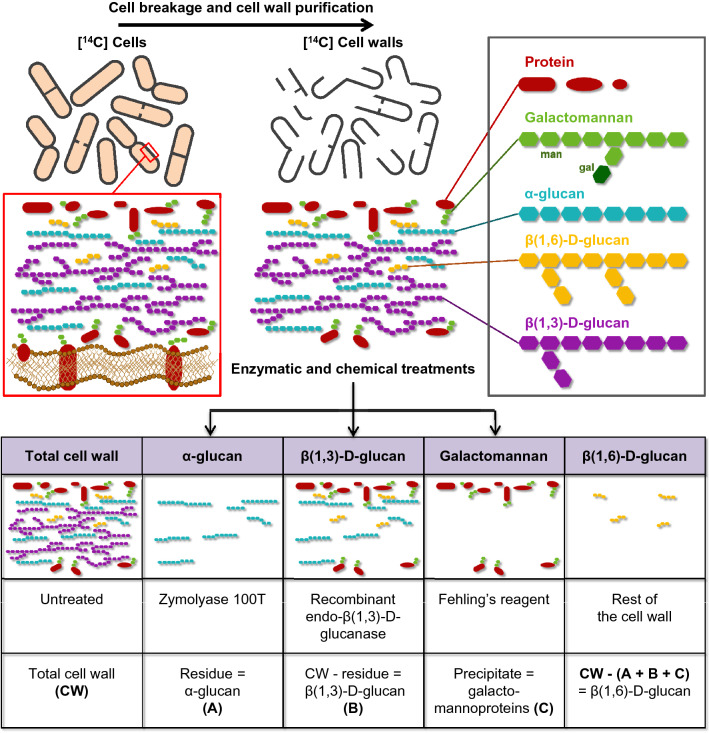
Top: Scheme of the [U-^14^C]-glucose radioactive labelling and fractionation of the polysaccharides of the fission yeast cell wall. A representation of the composition, organization and structure of the fission yeast cell wall is shown. Bottom: scheme of the enzymatic and chemical treatments used for the fractionation and analysis of the cell wall polysaccharides

We have previously described a simple and accurate method for analyzing the cell wall polymers of the fission yeast *Schizosaccharomyces pombe* by enzymatic and chemical analyses of radioactive labeled cell walls [16, 19–23]. Briefly, this protocol consists of ^14^C-glucose labelling and fractionation of cell wall polysaccharides by using specific chemical and enzymatic procedures (Fig. 1). This allows for the quick and accurate quantification of the main cell wall polymers: α-glucan, β(1,3)-d-glucan, β(1,6)-d-glucan and galactomannoproteins [22, 23]. Although this protocol has been established by using fission yeast cell walls, it can be easily adapted for analyzing the cell wall of other fungal species [24–28]. This protocol involves enzymatic degradation of the cell wall with the enzymatic complex Zymolyase 100T (Seikagaku Biobusiness Corporation) and the recombinant endo-β(1,3)-d-glucanase Quantazyme (MP Biomedicals), whose activities are essential for quantifying the amount of α-glucan and β(1,3)-d-glucan in the cell wall, respectively [22, 23]. To accomplish this, both enzymatic complex and recombinant enzyme must contain very precise and defined enzymatic activities, free from contaminants or unknown activities in the first case, and a completely specific and efficient activity in the second [15].

Zymolyase 100T is an enzymatic mixture partially purified from *Arthrobacter luteus*. It contains endo-β(1,3)-d-glucanase, protease and mannanase activities, but not α-glucanase activity [29, 30]. Thus, the cell wall residue after Zymolyase 100T degradation corresponds to the α-glucan (Fig. 1). Quantazyme is a recombinant endo-β(1,3)-d-glucanase from *Oerskovia xanthineolytica* (also known as *Cellulosimicrobium cellulans*) [31]. This glucanase does not contain either contaminant or unspecific activities and therefore, exclusively degrades the β(1,3)-d-glucan of the cell wall without affecting any other cell wall polysaccharides [16, 32]. Thus, the cell wall residue after Quantazyme degradation corresponds to the cell wall, except for β(1,3)-d-glucan (Fig. 1).

As the β(1,3)-d-glucan is a structural and essential polysaccharide of the cell wall, highly conserved in most fungi, Quantazyme has proven useful as a tool for the specific cell wall β(1,3)-d-glucan degradation and analysis in a variety of fungal genera [8–10, 18, 33–41]. According to the information provided by a previous supplier (Q-Biogene), many fungal genera (yeast and filamentous fungi) are susceptible to Quantazyme, either at low concentration (0.2 U/mL, *Ashbya, Endomyces, Kloeckera, Kluyveromyces, Pullularia, Saccharomyces*), at higher concentration (2.0 U/mL, *Candida, Debaryomyces, Eremothecium, Hansenula, Hanseniaspora, Lipomyces, Metschnikowia, Saccharomycopsis, Saccharomycodes, Schizosaccharomyces, Selenozyma, Trigonopsis, Wickerhamia*), or with strain-dependent variable susceptibility (*Brettanomyces, Cryptococcus, Nadsonia, Pichia, Rodosporidium, Schwanniomyces, Stephanoascus, Torulopsis*) showing a very similar lytic spectrum to that described for Zymolyase by its supplier. Thus, Quantazyme and non-recombinant endo-β(1,3)-d-glucanases have been widely used for studies on the cell wall, not only of yeast cells but also filamentous fungi and even plant cells [10, 33, 34, 42–47].

Endo-β(1,3)-d-glucanases have also been important for the genomic analyses of processes such as induced cell wall stress, altered cell wall integrity pathway and altered response to cell wall synthesis inhibitors [48–52]. In addition, endo-β(1,3)-d-glucanases have been frequently used for the analyses of cell wall composition, as well as enzymatic product required in the study of antifungal drugs and the discovery of new antifungals that specifically target the synthesis of cell wall polysaccharides [46, 53–59]

Despite all of the applications and uses of highly specific endo-β(1,3)-d-glucanases, Quantazyme was the only commercially available recombinant endo-β(1,3)-d-glucanase known to reproducibly and reliably degrade the fission yeast cell wall β(1,3)-d-glucan. Therefore, when this enzyme was discontinued it became extremely necessary to find a new recombinant endo-β(1,3)-d-glucanase with similar properties to replace Quantazyme in cell wall β(1,3)-d-glucan degradation studies [23, 24, 60]. Currently, some commercial enzymes described as endo-β(1,3)-d-glucanases may be useful as putative substitutes for Quantazyme; however, so far, none of them have been tested in the cell wall of *S. pombe* or any other fungi. Thus, the aim of this work was to identify those commercial recombinant enzymes exhibiting a specific and efficient β(1,3)-d-glucanase activity, free of any additional residual activity and able to replace Quantazyme in fission yeast cell wall analysis. For this purpose, we tested 12 commercially available recombinant endo-β(1,3)-d-glucanases and compared their activity to that of Quantazyme. Here, we show that GH16 enzyme from *Pyrococcus furiosus* (called PfLam16A) [61, 62], was able to perform a complete and reliable cell wall β(1,3)-d-glucan degradation similar to that of Quantazyme. Hence, the use of PfLam16A in future studies on the cell wall in either fission yeast or other fungal species in general appears to be quite promising.

## Results and discussion

The fungal cell wall is an essential structure that provides osmotic resistance and mechanical strength to fungal cells. It must be physically robust to withstand the force of turgor pressure within the cell. On the other hand, since it is also the interface between the fungal cell and the external environment, it must be a highly dynamic structure, capable of changing its composition and architecture in response to variable environmental conditions [3, 5]. Thus, to understand the mechanisms whereby fungi react to environmental conditions, it is necessary to have methods and tools to determine the cell wall composition throughout the life cycle of a cell [16].

In this work, we used the fission yeast as a model for studying cell wall composition [5], which has been described as a fast, simple and reliable tool for analyzing cell wall polysaccharides [63, 64]. The protocol employed has been improved over time with only some minor modifications. Initially, this protocol allowed the quantification of three cell wall polysaccharides, α-glucan, β-glucan (mixture of β(1,3)-d- and β(1,6)-d-glucans) and galactomannoproteins, and the percentage that each of them represents in both the cell wall and the cell [16, 19–21, 65] (Fig. 1). Later on, the protocol was improved in order to discriminate the different β-glucans and to quantify the percentage of each of the four main fungal cell wall polysaccharides: α-glucan, β(1,3)-d-glucan, β(1,6)-d-glucan and galactomannoproteins [14, 22, 23] (Fig. 1). Thus, the current protocol used for fission yeast cell wall fractionation involves three consecutive steps: (1) ^14^C-labelling of the cell; (2) isolation of cell wall; and (3) fractionation of the cell wall polysaccharides by using both enzymatic (Zymolyase 100T complex and recombinant β(1,3)-d-glucan-hydrolyzing enzyme called Quantazyme) and chemical (Fehling’s reagent) fractionations (Fig. 1). As mentioned above, since Quantazyme is no longer available, we sought to identify an alternative recombinant endo-β(1,3)-d-glucanase able to degrade the cell wall β(1,3)-d-glucan. Having said enzyme would not only permit degradation results similar to those achieved using Quantazyme, but would also ensure the continuity of studies on the fission yeast cell wall, as well as the cell walls of other fungi in general.

**Fig. 2:**
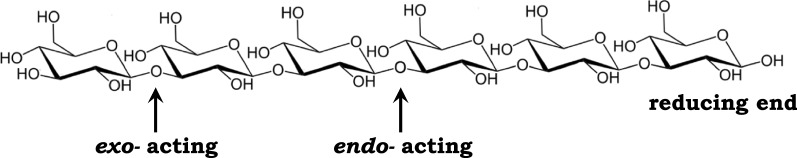
Sites of action of the different types of β(1,3)-d-glucan hydrolase enzymes in the β(1,3)-d-glucan chain. Exo-β(1,3)-d-glucanases cleave the terminal linkage releasing single glucose units form the non-reducing end of the β(1,3)-d-glucan chain. Endo-β(1,3)-d-glucanases cleave the internal linkages between glucose units along the β(1,3)-d-glucan chain releasing short oligosaccharides

Carbohydrate-active enzymes (CAZy) is a database that characterizes and groups structurally-related enzymes into different families based on the amino acid sequences of their structurally related catalytic or binding modules (www.cazy.org) [66–68]. Among them, glucan-hydrolyzing (GH) enzymes catalyze the hydrolytic cleavage of glycosidic bonds (Fig. 2). Each GH family contains proteins that are related, owing to their sequence and/or structure, which indicates a similar mechanism of action and a similar geometry around the glycosidic bond [66, 69, 70]. Therefore, this classification reveals the possible phylogenetic relationship between different families based on structural features rather than a relationship based on the efficiency of their endo-β(1,3)-d-glucanase activity [71]. According to the CAZy database, Quantazyme has been classified into family GH64 of glycosyl hydrolases [32]. The activity found in the GH64 family is the endo-hydrolysis of β(1,3)-d-glucosidic linkages in β(1,3)-d-glucans (Fig. 2). This activity is classified as E.C. (Enzyme Commission Number) 3.2.1.39 [60, 72, 73]. Other GH families with the same activity are GH5, GH16, GH17, GH55, GH64, GH81, GH128, GH152, GH157 and GH158 [71].

In addition, the β(1,3)-d-glucanase activity is also classified as E.C. 3.2.1.6, whose reaction is the endo-hydrolysis of β(1,3)-d- or β(1,4)-linkages in β-d-glucans when the glucose residue whose reducing group is involved in the linkage to be hydrolyzed is itself substituted at C-3 [73–76]. Another related activity classified as E.C. 3.2.1.73 consists of the hydrolysis of β(1,4)-d-glucosidic linkages in β-d-glucans containing (1,3) and (1,4) bonds [60, 68, 77, 78]. All of these enzymes are endo-hydrolases because they cleave internal linkages along the polysaccharide chain, releasing small oligosaccharides as hydrolysis products [69, 70] (Fig. 2).

The enzymes used in this study are classified according to their activity as E.C. 3.2.1.6, 3.2.1.39 and 3.2.1.73, and belong to the CAZy families GH16, GH17, GH55, GH64 and GH81 (see details on the classification of each enzyme in Additional file 1: Table S1, and on the production host, specific activity and purity in Additional file 2: Table S2) [31, 61, 62, 79–88]. Thus, we tested the activity of 12 recombinant enzymes described as specific endo-β(1,3)-d-glucanases from three different commercial suppliers. The cell wall degraded by Quantazyme was used as a control for comparing the amount of β(1,3)-d-glucan degraded using commercial enzymes (Table 1).

**Table 1:** Cell wall degradation by specific recombinant endo-β(1,3)-d-glucanases

Commercial suppliers	Recombinant enzyme	Reaction conditions					% of cell wall degradation	Source organism
		Specified by the supplier			Maximum degradation ^a^			
		Buffer	pH	Temp. (°C)	Incubation time (h)	Enzyme units or weight (µg)		
**MP Biomedicals**	**Quantazyme**	**Potassium phosphate monobasic/KOH 33.5 mM + β-mercaptoethanol 60 mM**	**7.5**	**37**	**25**	**400 U**^**b**^	**53.76 ± 1.29**	*Oerskovia xanthineolytica*
Megazyme	E-LAMHV	Sodium acetate 100 mM	5.0	40	25	100 U^c^	68.17 ± 0.15	*Hordeum vulgare* (barley)
	E-LICACT	Sodium phosphate 100 mM	6.5	60	36	120 U^d^	59.28 ± 0.20	*Clostridium thermocellum*
NZYTech	ALam55A	Sodium acetate 50 mM	5.0	45	25	50 µg	37.39 ± 2.04	*Arthrobacter* sp.
	BhLam81A	Sodium phosphate 50 mM	6.5	60	36	10 µg	17.59 ± 1.32	*Bacillus halodurans*
	CtLam81A	Sodium phosphate 50 mM	6.0	65	25	150 µg	54.37 ± 6.47	*Clostridium thermocellum*
	CtLic16A	MES 50 mM	6.0	65	36	35 µg	19.70 ± 6.18	*Clostridium thermocellum*
	**PfLam16A**	Sodium phosphate 100 mM	6.5	70	36	50 µg	**53.17 ± 2.56**	*Pyrococcus furiosus*
	TmLam16A	Sodium phosphate 50 mM	7.0	45	36	50 µg	34.44 ± 0.89	*Thermotoga maritima*
	TnLam16A	Sodium phosphate 50 mM	6.0	70	25	50 µg	66.75 ± 5.01	*Thermotoga neapolitana*
	TpLam16A	Sodium phosphate 50 mM	6.0	80	36	50 µg	68.25 ± 1.35	*Thermotoga petrophila*
	ZgLam16A	Glycine–NaOH 100 mM	8.5	40	36	10 µg	19.52 ± 0.36	*Zobellia galactanivorans*
Prokazyme	Bglu110	Sodium phosphate 100 mM	7.0	75	36	10 U^e^	78.23 ± 6.15	*Rhodothermus marinus*

^a^Reaction condition in which each enzyme exhibits the maximum % of cell wall degradation (mean ± SD from at least two independent experiments). In addition to these conditions, each enzyme was tested according to the conditions described in Additional file 3: Table S3

^b^One unit of Quantazyme activity is defined as the amount of enzyme required to produce a 0.001 decrease in A_800_ per minute from a suspension of brewer’s yeast (*Saccharomyces cerevisiae*) as substrate in 33.5 mM potassium phosphate monobasic buffer, pH 7.5 with KOH, 60 mM β-mercaptoethanol at 25 °C

^c^One unit of E-LAMHV activity is defined as the amount of enzyme required to release one µmole of glucose-reducing sugar equivalents per minute from laminarin β(1,3)-d-glucan (10 mg/mL) as substrate in 100 mM sodium acetate buffer, pH 5.0 at 40 °C

^d^One unit of E-LICACT activity is defined as the amount of enzyme required to release one µmole of glucose-reducing sugar equivalents per minute from barley β-d-glucan (5 mg/mL) as substrate in 100 mM sodium phosphate buffer, pH 6.5 at 40 °C

^e^One unit of Bglu110 activity is defined as the amount of enzyme required to release one µmole of glucose-reducing sugar equivalents per minute from lichenan β(1,3)(1,4)-d-glucan (10 mg/mL) as substrate in 100 mM sodium phosphate buffer, pH 7.0 at 75 °C

First, the endo-β(1,3)-d-glucanase activity for each enzyme was tested according to the conditions specified by the supplier. These conditions were specifically obtained through the enzymatic degradation of purified β(1,3)-d-glucan (laminarin or β(1,3)-d-glucan from barley) and β(1,3)(1,4)-glucan (lichenan), but not in the context of the fungal cell wall, where β(1,3)-d-glucan is closely intertwined with other polymers. Table 1 shows the list of recombinant enzymes tested, the percentage of cell wall degradation and the reaction conditions in which each enzyme exhibited maximum activity.

The recombinant enzymes ALam55A, BhLam81A, TmLam16A and ZgLam16A (from NZYTech) showed insufficient cell wall degradation, where the values obtained were well below from those obtained using Quantazyme, which was 53% of cell wall degradation. However, all of these enzymes belong to the same class of activity as Quantazyme, classified as E.C. 3.2.1.39 (Additional file 1: Table S1). Thus, the differences observed in the capacity of these enzymes to degrade could be due to their ability to access the β(1,3)-d-glucan substrate in the fission yeast cell wall, suggesting that the β(1,3)-d-glucan could likely be more or less inaccessible to the active site depending on the enzyme. Similarly, the E.C. 3.2.1.73 enzyme CtLic16A (also from NZYTech) was able to degrade a small fraction of the cell wall (Table 1). This enzyme hypothetically hydrolyzes β(1,4)-d-glucosidic linkages in β-d-glucans containing (1,3) and (1,4) bonds, however, the cell wall of fission yeast does not contain β(1,4)-glucan. Therefore, it is possible that this enzyme could contain some contamination or has additional activities that degrade or release polysaccharide fragments from the fission yeast cell wall. On the contrary, other recombinant enzymes, classified either as 3.2.1.39 (E-LICACT from Megazyme and TnLam16A or TpLam16A from NZYTech) or 3.2.1.6 (E-LAMHV from Megazyme and Bglu110 from Prokazyme) (Additional file 1: Table S1), showed excessive cell wall degradation, well above 53%, as compared to the Quantazyme control (Table 1). Despite being recombinant, these enzymes exhibited cell wall degradation that corresponded to the degradation of more than just one type of polysaccharide. Thus, these enzymes showed the signs of either containing contamination or having an activity that was stronger than that of Quantazyme. Alternatively, excessive β(1,3)-d-glucan degradation could cause the release of other cell wall polysaccharides that have never been detected using Quantazyme. Finally, the recombinant 3.2.1.39 enzymes PfLam16A and CtLam81A (NZYTech, Additional file 1: Table S1 and Additional file 2: Table S2) [61, 62, 82] exhibited optimal percentages of cell wall degradation that were similar to the control (Table 1). However, CtLam81A presented the disadvantage that it precipitated during the reaction, generating a rare viscous reaction mixture that was difficult to process and to quantify the efficacy of the degradation.

Next, in an attempt to improve the activity of low-efficient enzymes and to reduce the precipitation caused by CtLam81A, all enzymes were again tested under different reaction conditions (Additional file 3: Table S3). All combinations analyzed using different buffers, pH and incubation temperatures did not significantly alter the previous results obtained for ALam55A, BhLam81A, CtLic16A and ZgLam 16A, which were still degrading less than expected. In contrast, the recombinant enzymes E-LICACT, E-LAMHV, TmLam16A, TnLam16A and TpLam16A were highly sensitive to changes in buffer, pH and/or incubation temperature. They presented a broad range of cell wall degrading activities ranging from 8% (BhLam81A) to 73% (E-LAMHV) (Additional file 3: Table S3). This indicated that the activity of the enzymes assayed was not stable or reliable. Instead, they varied considerably when conditions other than those specified by the supplier were used. Therefore, these enzymes were not suitable for substituting Quantazyme in cell wall analysis. The stability and/or variability of the enzymes PfLam16A and CtLam81A were also analyzed, being two enzymes that had shown optimal percentages of cell wall degradation in previous experiments. While CtLam81A was extremely variable, with an activity that ranged from 14 to 81%, and still caused the reaction mixture to precipitate, PfLam16A, on the other hand, was found to be highly stable. The percentage of cell wall degradation was within the optimal range for all conditions tested, except when the reaction was carried out using decreasing amounts of enzyme or during incubation times that only allowed for partial cell wall degradation (Additional file 3: Table S3).

Then, the cell wall degradation was also analyzed by combining two enzymes simultaneously in the same reaction. The aim was to identify combinations capable of degrading the cell wall like PfLam16A or Quantazyme (Table 2). In all cases, a reaction condition was used in which the single enzyme, including PfLam16A, showed insufficient cell wall degradation. Under these conditions, some of the combinations still were unable to sufficiently degrade the cell wall, whereas others showed excessive cell wall degradation. The only enzyme combination that had the ability to degrade the cell wall to around 54% was that of CtLam81A and TmLam16A. Unfortunately, in this case the enzyme mixture again precipitated, making it difficult to handle the reaction.

**Table 2:** Percentage of cell wall degradation with combinations of two recombinant endo-β(1,3)-d-glucanase enzymes

Recombinant enzyme 1	Recombinant enzyme 2	Enzymes 1 + 2 Units or weight (µg)	Reaction conditions			% of cell wall degradation^a^
			Buffer	pH	Temp. (°C)	
E-LAMHV^b^	TmLam16A^c^	50 U + 50 µg	Sodium phosphate 50 mM	5.5	50	42.10 ± 0.09
E-LAMHV^b^	ZgLam16A^c^	50 U + 10 µg	Sodium phosphate 50 mM	6.5	40	41.69 ± 1.09
BhLam81A^c^	E-LAMHV^b^	10 µg + 50 U	Sodium phosphate 50 mM	5.5	50	44.51 ± 0.36
BhLam81A^c^	CtLam81A^c^	10 µg + 150 µg	Sodium phosphate 50 mM	6.5	60	33.87 ± 0.33
CtLam81A^c^	E-LAMHV^b^	150 µg + 50 U	Sodium phosphate 50 mM	5.5	50	67.77 ± 1.92
CtLam81A^c^	TmLam16A^c^	150 µg + 50 µg	Sodium phosphate 50 mM	6.5	60	54.80 ± 1.83
CtLam81A^c^	ZgLam16A^c^	150 µg + 10 µg	Sodium phosphate 50 mM	6.5	50	47.77 ± 7.28
CtLic16A^c^	E-LAMHV^b^	75 µg + 50 U	Sodium phosphate 50 mM	5.5	50	34.43 ± 0.37
CtLic16A^c^	CtLam81A^c^	75 µg + 150 µg	Sodium phosphate 50 mM	6.5	60	38.71 ± 3.63
PfLam16A^c^	TmLam16A^c^	40 µg + 50 µg	Sodium phosphate 100 mM	6.5	70	59.40 ± 3.43
PfLam16A^c^	TmLam16A^c^	40 µg + 50 µg	Sodium phosphate 50 mM	7.0	60	44.33 ± 1.04

^a^Percentage of cell wall degradation after 36 h of reaction (mean ± SD). All reactions were carried out from at least two independent experiments

^b^Commercial supplier: Megazyme

^c^Commercial supplier: NZYTech

Finally, and taking into account all previous results, recombinant PfLam16A was selected as the best enzyme. Subsequently, it was used to perform additional analyses before being selected as a substitute for Quantazyme (Table 3) [61, 62]. In order to determine the optimal conditions for cell wall degradation by PfLam16A, different conditions combining various parameters, such as the buffer used, pH, temperature, incubation time and the amount of enzyme, were assessed. In the case of sodium acetate (pH 5.0), a slight increase in cell wall degradation was detected at longer incubation times. This could be due to the fact that at acidic pH the cell wall can be partially hydrolyzed, helping the enzyme to release some oligosaccharides to the medium [89]. However, at a higher pH, undesirable acidic hydrolysis was prevented (Table 3). Most of the conditions tested were found not to be optimal, because cell wall degradation was insufficient. However, optimal cell wall degradation was achieved using the following buffers: citrate/phosphate 100 mM pH 5.5; MES 100 mM pH 5.5; sodium acetate 100 mM pH 5.0 and sodium phosphate 100 mM pH 6.0 and 6.5; and one specified by the supplier. All of these buffers, except sodium acetate pH 5.0, permitted stable and reproducible enzyme activity, with similar percentages of cell wall degradation being achieved at longer incubation times and with larger amounts of enzyme. Thus, the following conditions were established as the standard reaction (Table 3): 20 μg of PfLam16A in buffer sodium phosphate 100 mM pH 6.5 with an incubation of 20 h at 70 °C.

**Table 3:** Percentages of cell wall degradation with PfLam16A at different conditions

Buffer	pH	Temp. (^o^C)	Enzyme weight (µg)	Incubation time (h)	% of cell wall degradation ^a^
					Mean ± SD
Citrate/phosphate 100 mM	**5.5**	**70**	**20**	20	49.76 ± 0.18
				**25**	**51.86 ± 0.48**^**b**^
MES 100 mM	**5.5**	**70**	**20**	**20**	**54.79 ± 1.48**^**b**^
				**25**	**54.40 ± 1.48**^**b**^
	**5.5**	**70**	**40**	**20**	**50.64 ± 1.06**^**b**^
				**25**	**53.03 ± 0.36**^**b**^
	6.5	70	20	36	45.00 ± 0.11
Sodium acetate 100 mM	**5**	**70**	**5**	20	47.58 ± 0.05
				**25**	**51.55 ± 0.32**^**b**^
	**5**	**70**	**10**	**20**	**55.54 ± 0.21**^**b**^
				25	57.87 ± 0.49
	**5**	**70**	**20**	5	44.13 ± 1.44
				**10**	**52.50 ± 1.58**^**b**^
				**15**	**56.58 ± 0.53**^**b**^
				20	58.37 ± 1.82
				25	59.19 ± 2.82
				36	67.58 ± 0.21
	5	70	40	20	57.73 ± 3.30
				25	62.21 ± 4.01
Sodium phosphate 50 mM	6	60	20	15	33.25 ± 2.48
				20	33.39 ± 0.67
				25	39.17 ± 0.18
	6	70	20	15	39.67 ± 0.94
				20	44.84 ± 0.73
				25	48.90 ± 0.29
	6.5	60	20	15	30.53 ± 0.64
				20	31.95 ± 1.01
				25	37.53 ± 0.36
	6.5	70	20	15	38.89 ± 0.38
				20	42.68 ± 0.79
				25	45.38 ± 0.21
	7	70	20	15	41.59 ± 3.06
				20	39.82 ± 1.62
				25	41.14 ± 0.42
Sodium phosphate 100 mM	6	60	20	15 20 25	40.03 ± 0.98
					40.91 ± 1.28
					43.01 ± 0.22
	**6**	**70**	**20**	15 **20** **25**	45.57 ± 0.97
					**50.62 ± 3.59**^**b**^
					**52.34 ± 3.76**^**b**^
	**6**	**70**	**40**	**20** **25**	**51.57 ± 6.52**^**b**^
					**55.17 ± 5.78**^**b**^
	6.5	60	20	15	32.94 ± 2.08
				20	36.98 ± 2.05
				25	35.11 ± 0.86
	**6.5**	**70**	**20**	15	47.39 ± 3.99
				**20**	**52.80 ± 3.32**^**b,c**^
				**25**	**51.27 ± 2.79**^**b**^
	**6.5**	**70**	**40**	**15**	**51.01 ± 4.19**^**b**^
				**20**	**50.77 ± 0.28**^**b**^
				**25**	**51.32 ± 0.69**^**b**^
				**36**	**56.37 ± 2.33**^**b**^

^a^Percentage of cell wall degradation calculated from at least two independent experiments

^b^In bold are shown the values and conditions of percentage of cell wall degradation similar to that of Quantazyme (50–56%)

^c^Underlined is shown the selected condition for the standard protocol of cell wall degradation (lowest enzyme amount and incubation time using the reaction conditions specified by the supplier)

In order to confirm that PfLam16A and the reaction conditions established could achieve a similar percentage of cell wall degradation as that obtained using Quantazyme, separate reactions using PfLam16A and Quantazyme were carried out simultaneously (Table 4). As expected, both enzymes resulted in reproducible and highly similar percentages of cell wall degradation; thus, it was confirmed that PfLam16A [61, 62] was a definite substitute for Quantazyme for the study of cell wall β(1,3)-d-glucan degradation.

**Table 4:** Comparison between the enzymatic cell wall degradations with PfLam16A and Quantazyme

Enzyme	Enzyme units or weight (µg)^a^	% of cell wall degradation	Standard deviation (SD)
PfLam16A^b^	20 µg	53.63	± 2.22
Quantazyme^c^	400 units^d^	53.76	± 1.29

^a^Enzyme conditions (units or weight) provided by the commercial supplier

^b^The value is the average from seven independent experiments (mean ± SD). PfLam16A reaction conditions: 100 mM Sodium phosphate buffer, pH 6.5, 20 h, 70 °C. % of degradation in each of the seven experiments: 56.59%, 51.97%, 51.20%, 54.42%, 49.93%, 55.32%, 55.94%

^c^The value is the average from three independent experiments (mean ± SD). Quantazyme reaction conditions: 33.5 mM potassium phosphate monobasic/KOH buffer, pH 7.5, 60 mM β-mercaptoethanol, 24 h, 37 °C. % of cell wall degradation in each of the three experiments: 54.40%, 51.82%, 55.05%

^d^One unit of Quantazyme activity is defined as the amount of enzyme required to produce a 0.001 decrease in A_800_ per minute at pH 7.5 and 25 °C using a suspension of brewer’s yeast (*Saccharomyces cerevisiae*) as substrate

Finally, in order to show the usefulness of the proposed protocol, the complete fractionation and analysis of the cell wall polysaccharides of fission yeast was performed using recombinant endo-β(1,3)-d-glucanase PfLam16A (Table 5). As expected, the percentages obtained for each polysaccharide, including β(1,3)-d-glucan, were in agreement with those previously described for fission yeast using Quantazyme [16, 22, 23].

**Table 5:** Fractionation of the *S. pombe* cell wall polysaccharides using recombinant endo-β(1,3)-d-glucanase PfLam16A

Total cell wall^a^	
Polysaccharide	% of cell wall polysaccharide^b^
α-glucan Galactomannoprotein β-glucan^c^	28.60 ± 1.13 14.50 ± 1.40 56.90 ± 2.34
β(1,3)-d-glucan^c^ β(1,6)-d-glucan^c^	53.63 ± 2.22 3.28 ± 3.13

^a^The % of total cell wall in the cell is 35.74 ± 1.01

^b^Values are the average from three independent experiments (mean ± SD)

^c^β-glucan is the sum of β(1,3)-d-glucan plus β(1,6)-d-glucan

**Fig. 3:**
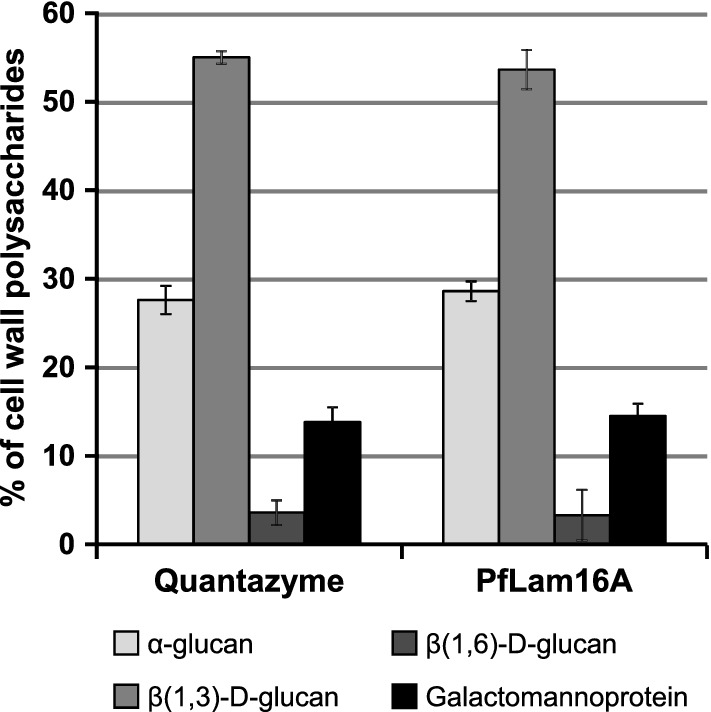
Comparison between complete cell wall fractionations using Quantazyme or PfLam16A. Cell wall fractionation data using Quantazyme are from previous work [92]. There is no significant difference between the data obtained from cell wall fractionations using Quantazyme or PfLam16A

In sum, a series of 12 commercial recombinant endo-β(1,3)-d-glucanases were analyzed, and PfLam16A was found to be the only enzyme that produced reliable and reproducible results. Moreover, PfLam16A was able to specifically and completely degrade cell wall β(1,3)-d-glucan of the fission yeast *S. pombe* without affecting other polysaccharides. Thus, our results show that this enzyme is the best suitable substitute for Quantazyme for the fractionation and analysis of cell wall polysaccharides from fission yeast and other fungi in general (Table 4; Fig. 3).

## Materials and methods

### Strains

The *Schizosaccharomyces pombe* strain used in this study was the wild type 972 h^−^.

### Growth media

The growth media used were YES (Yeast Extract with Supplements) and YES 0.5% Glc (YES with low glucose). Normal YES medium contains 30 g/L glucose, 5 g/L yeast extract, 250 mg/L adenine, 250 mg/L histidine, 250 mg/L leucine, 250 mg/L lysine and 250 mg/L uracil, sterilized by autoclaving. Alternatively, EMM (Edinburgh minimal medium) and EMM 0.5% Glc can also be used [90].

Carrier cells were grown in YEPD (Yeast Extract Peptone Dextrose) medium: 10 g/L yeast extract, 20 g/L bactopeptone and 20 g/L glucose, sterilized by autoclaving.

### Reagents

Buffers (each solution can be stored at room temperature for up to 6 months):

- Citrate/Phosphate (500 mM), pH 5.6: 71 g/L of Na_2_HPO_4_ + 96.1 g/L of citric acid, pH adjusted with 5 M NaOH.
- Glycine–NaOH (1 M), pH 8.5: 75.07 g/L of glycine, pH adjusted with 1 N NaOH.
- MES (2-(*N*-morpholino)ethanesulfonic acid) (1 M), pH 5.5, 6.0 and 6.5: 195.2 g/L of MES, pH adjusted with 5 M KOH.
- Potassium phosphate (1 M), pH 5.5, 7.0 and 7.5. Prepare 1 M stocks of each solution of K_2_HPO_4_ (174.2 g/L) and KH_2_PO_4_ (136.1 g/L). Adjust the pH by mixing them in different proportions as previously described [91].
- Sodium acetate (1 M), pH 5.0: 82 g/L of sodium acetate, pH adjusted with acetic acid.
- Sodium phosphate (1 M), pH 5.5, 6.0, 6.5 and 7.0: prepare 1 M stocks of each solution of Na_2_HPO_4_ (142.0 g/L) and NaH_2_PO_4_ (120.0 g/L). Adjust the pH by mixing the two solutions using different proportions as previously described [91].
- Tris–HCl (2-amino-2-hydroxymethyl-propane-1,3-diol) (1 M), pH 6.5 and 7.5: 121.1 g/L of Tris, pH adjusted with 1 N HCl.
- β-mercaptoethanol from Sigma Aldrich.
- d-[U-^14^C]glucose from Hartmann Analytic.
- EDTA (ethylenediaminetetraacetic acid) (1 M): 292.2 g/L of EDTA.
- Ethanol, absolute (≥ 99.8%).
- Fehling’s reagent: the reagent must be freshly prepared by adding Reagent B (3.5% CuSO_4_) to Reagent A (17.3% potassium sodium tartrate dissolved in 12.5% KOH) in a proportion of 1:1. Each reagent can be stored at room temperature for up to one year as long as reagent B is protected from light during storage.
- HCl (6 N): 511.1 mL/L of HCl
- Liquid scintillation cocktail from PerkinElmer.
- NaCl (5 M): 292.2 g/L of NaCl.
- NaN_3_ (sodium azide; 0.2%).
- NaOH (12%).
- Trichloroacetic acid (10%).
- Yeast mannan from Sigma Aldrich, (stock solution of 200 mg/mL in water, stored at – 20 °C).
- Zymolyase 100T from Seikagaku Biobusiness Corporation. Enzymatic mixture partially purified from *Arthrobacter luteus* with β(1,3)glucanase, protease and mannanase activities but not α-glucanase activity [29, 30]. Stock solution at 5 mg/mL in 50 mM citrate/phosphate buffer, pH 5.6 (standard reaction contains 5 µL, or 25 μg).
- Quantazyme from MP Biomedicals (discontinued): a recombinant endo-β(1,3)glucanase from *Oerskovia xanthineolytica* that specifically degrades β(1,3)-d-glucan without contaminating activity [31]. Stock solution at 20,000 units/mL in 33.5 mM potassium phosphate monobasic/KOH + 60 mM β-mercaptoethanol buffer, pH 7.5 (standard reaction contains 20 µL, or 400 units).
- Other recombinant enzymes, with endo-β(1,3)-d-glucanase activity, used in this work and their suppliers (NZYTech, Megazyme and Prokazyme) are shown in Table 1. Stock solutions vary in a range of 0.25–3.0 mg/mL or 1000–2100 units/mL in the corresponding buffer, according to the recommendations specified by the supplier. The buffer and amount of enzyme used in each reaction is indicated in each table.

### Fractionation and analysis of cell wall polysaccharides

This protocol has been adapted to quantify the three major polysaccharides of the *S. pombe* cell wall (α-glucans, β-glucans, and galactomannoproteins) by using new commercially available recombinant endo-β(1,3)-d-glucanases (from NZYTech, Megazyme and Prokazyme) and is based on the enzymatic and chemical fractionation of cell wall polysaccharides. Although the new protocol is essentially the same as one previously described [14, 16, 22, 23], it now includes information describing the search for a new recombinant endo-β(1,3)-d-glucanase that is both efficient and reliable, and able to replace Quantazyme (MP Biomedicals), which is no longer commercially available. Quantazyme is the only recombinant endo-β(1,3)-d-glucanase known to date that can completely and reproducibly degrade *S. pombe* cell wall β(1,3)-d-glucan. Since we have shown that the new recombinant endo-β(1,3)-d-glucanase PfLam16A (NZYTech) has a similar activity to that of Quantazyme, this protocol can be used for the analysis of any fungal cell wall. Briefly, the fractionation and analysis of the polymer composition of the cell wall is carried out in three main steps: (A) ^14^C-glucose labeling of the cells; (B) cell breakage and purification of the cell wall; and (C) fractionation and analysis of cell wall polysaccharides.

### (A) ^14^C-glucose labeling of the cells

1. Prepare a stock of unlabeled wild-type cells using the following method. These cells will be used as carrier cells in Step 9:
  i. Incubate wild-type cells in 500 mL of YEPD at 28ºC, with shaking at 200 rpm for 48 h, until late stationary phase.
  ii. Collect the cells by centrifugation at 4000*g* for 5 min, wash the pellet with distilled water, recentrifuge, and resuspend the cell pellet in 50 mL of 1 mM EDTA, 0.02% NaN_3_ (sodium azide). The cell suspension can be stored at 4 °C for up to 12 months and the final cell concentration is usually around 10^10^ cells/mL. Carrier cells are added in Step 9, described below, to minimize the loss of either ^14^C-labeled cells or cell walls during the different centrifugation steps.
2. Prepare cells in early log-phase in liquid medium (in the case of fission yeast, YES rich medium or EMM minimal medium) by growing, with shaking, the culture at the same temperature as that described below for the medium containing d-[U-^14^C]glucose. The medium should contain 0.5% or 1.0% of glucose (normal YES contains 3% and EMM contains 2%) in order to increase the efficiency of the subsequent incorporation of ^14^C-glucose into the cell.
3. Dilute the cells in 14 mL of the same medium, calculating the appropriate dilution in order to collect the cells in early log-phase at 1.0 to 1.5 X 10^7 ^cells/mL (A_600_ = 0.7–1.0).
4. Transfer the 14-mL cell culture to two new flasks (7 mL each): One culture will be used to monitor cell growth (unlabeled), and 3 µCi/mL of d-[U-^14^C]glucose (Hartmann Analytic) will be added to the other. When required, the concentration of ^14^C-glucose can be increased to 10 or 20 µCi/mL.
5. Incubate both cultures at the desired temperature with shaking. Use an incubation time that allows ^14^C to efficiently incorporate into the cells, taking into consideration that during each cell cycle, 50% of the cell material is newly synthesized and consequently ^14^C-labeled. A greater amount of labeling can be obtained using longer incubation times that allow more cell cycles to occur, or by increasing the concentration of ^14^C-glucose in the medium. Use the unlabeled cell culture to monitor cell growth.
6. When the monitored unlabeled cells have reached the desired absorbance (1.0 to 1.5 × 10^7 ^cells/mL), transfer the labeled cells to a 10 mL centrifuge tube and collect by centrifugation (at 4000*g* for 10 min).
7. Carefully discard the supernatant in the appropriate radioactive container; save some supernatant so to avoid losing any cells.
8. Wash the cells twice to eliminate the residual radioactive medium with 10 mL of 1 mM EDTA and centrifuge after each wash at 4000*g* for 10 min.
9. Resuspend the labeled cells in 10 mL of 1 mM EDTA and add 150 µL of carrier cells (prepared according to Step 1). Centrifuge, wash with 1 mM EDTA and then spin again at 4000*g* for 10 min.
  If the carrier cells are added before eliminating the remaining radioactive medium, they can incorporate some ^14^C-glucose that may interfere with the final result.
10. Resuspend the cells in a final volume of 1.1 mL of 1 mM EDTA and transfer them to a 1.5 mL screw-cup centrifuge tube.
11. Take two 50-µL aliquots of cells and mix with 2 mL of liquid scintillation cocktail in liquid scintillation vials. Mix by vortexing and keep at 4 °C until the amount of radioactivity is measured together with the other fractions (see Step 27 below). The radioactivity in these aliquots corresponds to the total amount of 14C-glucose incorporated into the cell fraction.

### (B) Cell breakage and purification of the cell wall

12. Centrifuge the remaining 1.0 mL of cells at 5000*g* for 3 min and discard the supernatant (the remaining drops are kept to avoid cell loss). Then, add 100 µL of 1 mM EDTA and resuspend the pellet to homogeneity using a vortex.
13. Add glass beads (0.5 mm diameter) to completely cover the cell suspension. Break the cells in a cell disrupter FastPrep FP120 (MP Biomedicals, Thermo Scientific): 3 pulses of 20 s each at a speed of 6.0 and a temperature of 4 °C. Check for complete cell breakage by microscopic observation (discard the glass slide in the radioactive waste).
14. Transfer the cell debris and glass beads to a 10 mL tube (Tube A) by adding 500 µL of 1 mM EDTA, vortexing and pouring the content into the new tube. Repeat the process until the microtube is completely clean. Top up the volume to 7 mL with 1 mM EDTA.
15. Vortex Tube A to dilute the cell debris in the 7 mL of 1 mM EDTA. The glass beads will quickly concentrate at the bottom of the tube. Carefully transfer the supernatant to a new 10 mL tube (Tube B). Centrifuge Tube B (at 4000*g* for 10 min), discard the supernatant in the appropriate radioactive container and repeat this process (see Step 16).
16. Add 7 mL of 1 mM EDTA to Tube A containing the glass beads to wash the beads and to recuperate all of the cell walls. As before, vortex Tube A and transfer the supernatant to Tube B. Centrifuge Tube B, discard the supernatant and repeat these steps at least 3 times or until the supernatant is transparent. This will help to collect all of the cell walls and to remove a considerable amount of cell debris.
17. Wash the pellet twice with 5 M of NaCl by centrifugation at 4000*g* for 10 min to separate the rest of cell membranes from the cell walls.
18. Wash the pellet three times with 1 mM of EDTA by centrifugation at 4000*g* for 5 min to eliminate the NaCl and some of the other residues.
19. Resuspend the cell wall pellet in 250 μL of 1 mM EDTA. Transfer it to a screw-cup 1.5 mL tube. Wash Tube B three times with 250 μL of 1 mM EDTA and transfer the contents to the 1.5 mL tube, and mix well. Adjust the final volume to 1.1 mL with 1 mM EDTA.
20. Heat the microtube in a thermoshaker for 20–30 min at 100 °C to deactivate the glucanases. Cool the tube for 10 min at 4 °C, briefly spin the tube and mix the content to homogeneity using a vortex.
21. Take two 50-µL aliquots of cell walls and mix with 2 mL of liquid scintillation cocktail in liquid scintillation vials. Vortex and keep at 4 °C until measuring its radioactivity together with the other fractions (see Step 27 below). The radioactivity in these aliquots corresponds to the total incorporation in the cell wall fraction.
22. To inhibit the growth of undesirable aerobic microorganisms that might consume the isolated cell wall, add 10 µL of 2% NaN_3_ (sodium azide) to the remaining 1.0 mL of cell wall suspension, mix well and store the cell walls at 4 °C for up to 2 weeks.

### (C) Fractionation and analysis of the cell wall polysaccharides:

Once the cell walls are ^14^C-labeled and isolated from the rest of cellular components, proceed to fractionate the cell wall polysaccharides as follows:

23. Cell wall degradation with Zymolyase 100 T:
  - Prepare a stock solution of Zymolyase 100 T at 5 mg/mL in 50 mM citrate/phosphate buffer, pH 5.6. The stock can be stored at – 20 °C for several years.
  - Transfer four 50-µL aliquots of cell walls to 1.5 mL tubes.
  - Add 30 µL of 0.5 M citrate/phosphate buffer, pH 5.6 to each tube. This buffer is 10 × concentrated and the final buffer concentration in the reaction mixture will be 50 mM.
  - Add 5 µL of the stock solution of Zymolyase 100 T (from the Step 23a above) into two of the tubes. Add 5 µL of distilled water into the other two tubes, which will be used as controls (no enzymatic degradation).
  - Top up the volume in each tube to 300 µL with distilled water and mix briefly by vortexing.
  - Incubate for 24 h at 37 °C with shaking in a thermoshaker or a roller apparatus.
  - Stop the reaction by adding 700 µL of 14.3% TCA (to make 10% TCA in a final volume of 1 mL).
  - Store the tubes at 4 °C until all samples are ready to be processed together (Step 25).
24. Cell wall degradation with recombinant endo-β-(1,3)-d-glucanase:
  - Transfer four 50-µL aliquots of cell walls to 1.5 mL tubes.
  - Add 30 µL of the required buffer at a concentration of 10 ×. In the case of PfLam16A, 1 M sodium phosphate buffer, pH 6.5 is the optimal 10 × buffer to use. In the case of Quantazyme add 30 µL of 10 × buffer (10 × is 335 mM potassium phosphate monobasic, pH 7.5 with KOH) and 30 µL of 10 × β-mercaptoethanol (10 × is 600 mM β-mercaptoethanol). The final 1 × buffer contains 33.5 mM potassium phosphate monobasic, pH 7.5 with KOH and 60 mM β-mercaptoethanol.
  - Add from 10 to 400 units or from 5 to 300 µg of the corresponding endo-β(1,3)-d-glucanase into two of the tubes. This corresponds to a volume from 5 to 100 µL of the corresponding stock solution of recombinant endo-β(1,3)-d-glucanase (prepared in the corresponding buffer as specified by the supplier). Add the same volume, from 5 to 100 µL of distilled water, into the other two tubes, which will be used as the controls (no enzymatic degradation). Table 1 lists all of the recombinant endo-β(1,3)-d-glucanases used in this work. In the case of PfLam16A, the optimal enzyme concentration is 20 µg per reaction.
  - Top up the volume in each tube to 300 µL with distilled water and mix briefly by vortexing.
  - Incubate with shaking in a thermoshaker or a roller in the conditions specified by the supplier for each enzyme. In the case of PfLam16A, the optimal condition is an incubation time of 20 h at 70 °C.
    Each tested enzyme has an optimal condition according to the specifications of the supplier (buffer, pH, temperature).
    The enzymes exhibiting a good percentage of cell wall degradation (about 55%, which is like that of Quantazyme, or slightly lower) were then tested under different conditions in order to assess their stability and to determine their maximum capacity to degrade cell walls.
  - Stop the reaction by adding 700 µL of 14.3% TCA (to make 10% TCA and a final volume of 1 mL).
  - Store the tubes at 4 °C until all samples are ready to be processed together (Step 25).
25. Process the enzymatic cell wall degradations carried out in Steps 23 and 24:
  - Spin the tubes. Filter the samples through glass microfiber filters discs (Whatman, grade GF/C) by vacuum filtration. The filter should be moistened with 10% TCA before adding the sample.
  - Wash the filter once with 1 mL of 10% TCA and once with 2 mL of absolute ethanol using vacuum filtration.
  - Insert the filter into a liquid scintillation vial and add 2 mL of liquid scintillation cocktail. Make sure the filter is completely covered by the liquid. Keep the vials at 4 °C until measuring their radioactivity (Step 27).
  - Fractionation of the galactomannoproteins:
    - Transfer two 250-µL aliquots of cell walls (from Step 22) to 1.5 mL tubes (it is advisable to use screw cap tubes). Add 250 µL of 12% NaOH (3 M) and heat for 60 min at 80 °C with shaking in a thermoshaker. Cool the tubes on ice for 15 min.
    - Centrifuge at 15,000*g* for 10 min. This step will eliminate any residual insoluble material that could interfere with galactomannan quantification.
    - Transfer 400 µL of the supernatant into new 10 mL tubes and add 20 µL of 200 mg/mL (4 mg) of yeast mannan as the carrier, and mix well.
    - Slowly add 2 mL of Fehling’s reagent and vortex the tubes gently. Store the reactions at 4 °C for 15 h (or overnight); this step will precipitate the galactomannan.
    - Centrifuge at 4000 g for 10 min and discard the supernatant by decanting. Wash the pellet twice with 3 mL of Fehling’s reagent (vortex gently) by centrifuging at 4000 g for 10 min.
    - Solubilize the pellet by slowly adding drop by drop 15–20 µL of 6 N HCl. Vortex the samples gently until solubilization in order to add the lowest volume of HCl as possible.
    - Add 100 µL of 50 mM Tris–Hcl pH 7.5, and mix well. Transfer and mix with 2 mL of liquid scintillation cocktail already present in scintillation vials.
    - Repeat the previous step twice, adding 100 µL of 50 mM Tris–HCl pH 7.5 to wash the tube. Transfer the solution to the corresponding liquid scintillation vial, and mix well.
26. Measure the radioactivity of the fractions.
  Measure the radioactivity of the liquid scintillation vials of each fraction in a liquid scintillation counter.
27. Analysis of the cell wall polysaccharides.
  Normalize the radioactivity according to the volume used in each sample in order to calculate the counts in the total volume of each fraction (Fig. 1):
  - Cells: average counts from the two tubes (Step 11) × 1000/50 = Counts of total cells.
  - Cell walls: average counts from the two tubes (Step 21) × 1100/50 = Counts of total cell walls.
  - α-glucan: average counts from the two tubes of Zymolyase 100 T degradation (Step 23) × 1100/50 = Counts of total α-glucan.
  - Galactomannoproteins: average counts from the two tubes of Feling’s reagent precipitation (Step 26) × 1100/250 × 500/400 = Counts of total galactomannoproteins.
  - β-glucan: average counts of the cell wall (Step 28b) minus the counts of [α-glucan + galactomannoproteins] = Counts of total β-glucan.
    β(1,3)-d-glucan: average counts of the cell wall (Step 28b) minus the average counts of the cell wall after recombinant endo-β(1,3)-d-glucanase degradation (Step 24).
    β(1,6)-d-glucan: average counts of the cell wall (Step 28b) minus counts of [α-glucan + β(1,3)-d-glucan + galactomannoproteins].

Each polysaccharide fraction can be expressed as a percentage of the radioactivity in the cell, according to the total amount of glucose incorporated into the cell, or as a percentage of the radioactivity in the cell wall, according to the total amount of radioactivity in the cell wall (the sum of all fractions is the total amount of the cell wall, which is 100%). The first set of data is the total amount of each polysaccharide relative to the cell and the second one shows the proportion of each polysaccharide with respect to the cell wall. The analysis of these percentages in different altered yeast strains may reveal defects in the proportion with respect to either the cell, or the cell wall, or both structures.

## Supplementary Information

**Additional file 1**: **Table S1**. Information about the tested recombinant endo-β(1,3)-d-glucanases

**Additional file 2**: **Table S2**. Characteristics of the tested recombinant endo-β(1,3)-d-glucanases.

**Additional file 3**: **Table S3**. Additional conditions tested for each enzyme

## Data Availability

All the recombinant endo-β(1,3)-d-glucanases and the enzymatic complex Zymolyase 100T used in this study are commercially available, as specified in the “Material and Methods” section.
