# Supplementary material for: Analysis and application of a suite of recombinant endo-β(1,3)-d-glucanases for studying fungal cell walls
Source: Microb Cell Fact. 2021 Jul 3;20:126. doi: 10.1186/s12934-021-01616-0 (PMC8254974; doi:10.1186/s12934-021-01616-0)
Supplement: Supplementary file 1 — Additional file 1: Table S1. Information about the tested recombinant endo-β(1,3)-d-glucanases [file 12934_2021_1616_MOESM1_ESM.docx]

**Table S1. Information about the tested recombinant endo-β(1,3)-D-glucanases**

| **Commercial suppliers** | **Recombinant enzyme** | **CAZy** ^a^ **family** | **E. C. number** ^b^ | **Source organism** |
| --- | --- | --- | --- | --- |
| MP Biomedicals | Quantazyme | GH 64 | 3.2.1.39 | *Oerskovia xanthineolytica* |
|  |  |  |  |  |
| Megazyme | E-LAMHV | GH 17 | 3.2.1.39 | *Hordeum vulgare* (barley) |
|  | E-LICACT | GH 16 | 3.2.1.6 | *Clostridium thermocellum* |
|  |  |  |  |  |
| NZYTech | ALam55A | GH 55 | 3.2.1.39 | *Arthrobacter* sp. |
|  | BhLam81A | GH 81 | 3.2.1.39 | *Bacillus halodurans* |
|  | CtLam81A | GH 81 | 3.2.1.39 | *Clostridium thermocellum* |
|  | CtLic16A | GH 16 | 3.2.1.73 | *Clostridium thermocellum* |
|  | PfLam16A | GH 16 | 3.2.1.39 | *Pyrococcus furiosus* |
|  | TmLam16A | GH 16 | 3.2.1.39 | *Thermotoga maritima* |
|  | TnLam16A | GH 16 | 3.2.1.39 | *Thermotoga neapolitana* |
|  | TpLam16A | GH 16 | 3.2.1.39 | *Thermotoga petrophila* |
|  | ZgLam16A | GH 16 | 3.2.1.39 | *Zobellia galactanivorans* |
|  |  |  |  |  |
| Prokazyme | Bglu110 | GH 16 | 3.2.1.6 | *Rhodothermus marinus* |

a. CAZy: database of **c**arbohydrate-**a**ctive en**zy**mes (CAZymes).

b. E.C. number: Enzyme Commission number
