# Supplementary material for: Analysis and application of a suite of recombinant endo-β(1,3)-d-glucanases for studying fungal cell walls
Source: Microb Cell Fact. 2021 Jul 3;20:126. doi: 10.1186/s12934-021-01616-0 (PMC8254974; doi:10.1186/s12934-021-01616-0)
Supplement: Supplementary file 2 — Additional file 2: Table S2. Characteristics of the tested recombinant endo-β(1,3)-d-glucanases. [file 12934_2021_1616_MOESM2_ESM.docx]

**Table S2. Characteristics of the tested recombinant endo-β(1,3)-D-glucanases**

**Recombinant enzyme Production host** ^a^ **Specific activity** ^a^ **Electrophoretic purity** ^a^

Quantazyme  *Escherichia coli* 20,000 U/mL ^b^ Purified to near homogeneity. Single band on SDS-PAGE followed by Coomassie blue staining (Mw 57 kDa)

E-LAMHV Not specified 100 U/mg ^c^ High purity. Single band on SDS-PAGE followed by Coomassie blue staining 2,100 U/mL (Mw 34 kDa); single major band on isoelectric focusing (pI 8.4)

E-LICACT Not specified 186 U/mg ^d^ High purity. Single band on SDS-PAGE followed by Coomassie blue staining 1,250 U/mL (Mw 29 kDa); single major band on isoelectric focusing (pI 4.9)

ALam55A *Escherichia coli* Not specified ^e^ Protein purity >90% as judged by SDS-PAGE followed by BlueSafe staining

(Mw 65 kDa)

BhLam81A *Escherichia coli* 163 U/mg ^f^ Protein purity >90% as judged by SDS-PAGE followed by BlueSafe staining

(Mw 87 kDa)

CtLam81A *Escherichia coli* 1,500 U/mg ^g^ Protein purity >90% as judged by SDS-PAGE followed by BlueSafe staining

(Mw 82 kDa)

CtLic16A *Escherichia coli* 9,000 U/mg ^h^ Protein purity >90% as judged by SDS-PAGE followed by BlueSafe staining

(Mw 27 kDa)

PfLam16A *Escherichia coli* 1,073 U/mg ^i^ Protein purity >90% as judged by SDS-PAGE followed by BlueSafe staining

(Mw 34 kDa)

TmLam16A *Escherichia coli* Not specified ^j^ Protein purity >50% as judged by SDS-PAGE followed by BlueSafe staining

(Mw 32 kDa)

TnLam16A *Escherichia coli* 2,600 U/mg ^k^ Protein purity >50% as judged by SDS-PAGE followed by BlueSafe staining

(Mw 33 kDa)

TpLam16A *Escherichia coli* 261 U/mg ^l^ Protein purity >50% as judged by SDS-PAGE followed by BlueSafe staining

(Mw 31 kDa)

ZgLam16A *Escherichia coli* Not specified ^m^ Protein purity >90% as judged by SDS-PAGE followed by BlueSafe staining

(Mw 31 kDa)

Bglu110 *Escherichia coli* 400 U/mg ^n^ Protein purity >95%. Single band on SDS-PAGE followed by Coomassie blue staining (Mw 32 kDa)

a. Information provided by the suppliers.

b. One unit of Quantazyme activity is defined as the amount of enzyme required to produce a 0.001 decrease in A_800_ per minute from a suspension of brewer’s yeast (*Saccharomyces cerevisiae*) as substrate in 33.5 mM potassium phosphate monobasic buffer, pH 7.5 with KOH, 60 mM β-mercaptoethanol at 25^o^C.

c. One unit of E-LAMHV activity is defined as the amount of enzyme required to release one µmole of glucose-reducing sugar equivalents per minute from laminarin β(1,3)-D-glucan (10 mg/mL) as substrate in 100 mM sodium acetate buffer, pH 5.0 at 40^o^C.

d. One unit of E-LICACT activity is defined as the amount of enzyme required to release one µmole of glucose-reducing sugar equivalents per minute from barley β-D-glucan (5 mg/mL) as substrate in 100 mM sodium phosphate buffer, pH 6.5 at 40*^o^C*.

e. ALam55A optimal enzymatic activity at pH 5.0 and 45^o^C.

f. One unit of BhLam81A activity is defined as the amount of enzyme required to release one µmole of glucose-reducing sugar equivalents per minute from laminarin β(1,3)-D-glucan as substrate. Optimal enzymatic activity at pH 6.0-8.0 and 60^o^C.

g. One unit of CtLam81A activity is defined as the amount of enzyme required to release one µmole of glucose reducing-sugar equivalents per minute from laminarin β(1,3)-D-glucan as substrate in 50 mM phosphate buffer, pH 6.0 at 60^o^C.

h. One unit of CtLic16A activity is defined as the amount of enzyme required to release one µmole of glucose reducing-sugar equivalents per minute from barley β-D-glucan as substrate in 50 mM MES buffer, pH 6.0 at 60^o^C.

i. One unit of PfLam16A activity is defined as the amount of enzyme required to release one µmole of reducing-sugar equivalents per minute from laminarin β(1,3)-D-glucan as substrate in 100 mM phosphate buffer, pH 6.5 at 80^o^C.

j. TmLam16A optimal enzymatic activity at pH 7.0 and 45^o^C.

k. One unit of TnLam16A activity is defined as the amount of enzyme required to release one µmole of reducing-sugar equivalents per minute from laminarin β(1,3)-D-glucan as substrate in 50 mM sodium phosphate/citrate buffer, pH 6.2 at 85^o^C.

l. One unit of TpLam16A activity is defined as the amount of enzyme required to release one µmole of reducing-sugar equivalents per minute from laminarin β(1,3)-D-glucan as substrate at pH 6.0 and 91^o^C.

m. ZgLam16A optimal enzymatic activity at pH 8.5 and 40^o^C.

n. One unit of Bglu110 activity is defined as the amount of enzyme required to release one µmole of glucose-reducing sugar equivalents per minute from lichenan β(1,3)(1,4)-D-glucan (10 mg/mL) as substrate in 100 mM sodium phosphate buffer, pH 7.0 at 75^o^C.
