# Supplementary material for: Analysis and application of a suite of recombinant endo-β(1,3)-d-glucanases for studying fungal cell walls
Source: Microb Cell Fact. 2021 Jul 3;20:126. doi: 10.1186/s12934-021-01616-0 (PMC8254974; doi:10.1186/s12934-021-01616-0)
Supplement: Supplementary file 3 — Additional file 3: Table S3. Additional conditions tested for each enzyme [file 12934_2021_1616_MOESM3_ESM.docx]

**Table S3. Additional conditions tested for each enzyme**

| **Recombinant enzyme** | **Reaction conditions** | | | | | **Range of % of cell wall degradation** |
| --- | --- | --- | --- | --- | --- | --- |
|  | **Incubation time (h)** | **Enzyme units**  **or weight (µg)** | **Buffer** | **pH** | **Temp. (^o^C)** |  |
| E-LAMHV | 25, 36 | 10, 20, 50, 100 U ^a^ | Sodium acetate 50 mM | 5.0 | 37, 40 | 29-55 |
|  | 36 | 100, 200 U | Sodium acetate 100 mM | 5.0 | 40 | 48-68 |
|  | 15, 20, 25, 36 | 10, 20, 50, 100 U | Sodium phosphate 50 mM | 5.5, 6.0, 6.5 | 40, 50 | 33-73 |
|  | 15, 20, 25 | 50 U | Sodium phosphate 100 mM | 6.5 | 70 | 71-73 |
|  |  |  |  |  |  |  |
| E-LICACT | 36 | 30, 60, 120 U ^b^ | Sodium phosphate 50 mM | 6.5 | 60 | 31-59 |
|  | 25, 36 | 60, 120 U | Sodium phosphate 100 mM | 6.5 | 60 | 40-50 |
|  |  |  |  |  |  |  |
| ALam55A | 25 | 50, 100 µg | Sodium acetate 50 mM | 5.0 | 37, 45 | 25-37 |
|  | 25 | 50, 100 µg | MES ^c^ 50 mM | 5.5 | 45 | 28-30 |
|  | 25 | 50, 100 µg | Potassium phosphate 50 mM | 5.5, 7.5 | 37, 45 | 22-31 |
|  |  |  |  |  |  |  |
| BhLam81A | 25, 36 | 5, 10 µg | Potassium phosphate 50 mM | 7.0 | 60 | 8-15 |
|  | 36 | 10 µg | Sodium phosphate 50 mM | 5.5, 6.5 | 50, 60 | 16-18 |
|  |  |  |  |  |  |  |
| CtLam81A ^d^ | 5, 10, 15, 20, 25, 36 | 15, 30, 75, 150, 300 µg | Sodium phosphate 50 mM | 5.5, 6.0, 6.5, 7.0 | 50, 60, 65 | 14-75 |
|  | 15, 20, 25 | 150 µg | Sodium phosphate 100 mM | 6.5 | 70 | 66-81 |
|  |  |  |  |  |  |  |
| CtLic16A | 36 | 35, 75, 150 µg | MES ^c^ 50 mM | 6.0 | 65 | 10-20 |
|  | 36 | 75 µg | Sodium phosphate 50 mM | 5.5, 6.5 | 50, 60 | 11-16 |
|  |  |  |  |  |  |  |
| **PfLam16A** | 20, 25 | 20 µg | Citrate/Phosphate 100 mM | 5.6 | 70 | 49-52 |
|  | 20, 25, 36 | 20, 40 µg | MES ^c^ 100 mM | 5.5, 6.5 | 70 | 45-55 |
|  | 5, 10, 15, 20, 25, 36 | 5, 10, 20, 40 µg | Sodium acetate 100 mM | 5.0 | 70 | 44-67 |
|  | 15, 20, 25 | 20 µg | Sodium phosphate 50 mM | 6.0, 6.5, 7.0 | 60, 70 | 30-48 |
|  | 15, 20, 25, 36 | 20, 40 µg | Sodium phosphate 100 mM | 6.0, 6.5 | 60, 70 | 32-55 |
|  |  |  |  |  |  |  |
| TmLam16A | 25, 36 | 20, 50 µg | Sodium phosphate 25  mM | 7.0 | 75 | 17-23 |
|  | 15, 20, 25, 36 | 20, 50, 100 µg | Sodium phosphate 50 mM | 5.5, 6.5, 7.0 | 45, 50, 60 | 31-62 |
|  | 15, 20, 25 | 50 µg | Sodium phosphate 100 mM | 6.5 | 70 | 54-64 |
|  |  |  |  |  |  |  |
| TnLam16A | 15, 20, 25, 36 | 20, 50 µg | Sodium phosphate 50 mM | 6.0, 7.0 | 60, 70 | 43-67 |
|  | 15, 20, 25 | 50 µg | Sodium phosphate 100 mM | 6.5 | 70 | 56-63 |
|  |  |  |  |  |  |  |
| TpLam16A | 15, 20, 25, 36 | 10, 20, 50 µg | Sodium phosphate 50 mM | 6.0 | 70, 80 | 51-68 |
|  | 15, 20, 25 | 50 µg | Sodium phosphate 100 mM | 6.5 | 70 | 57-61 |
|  | 25, 36 | 50 µg | Tris-HCl ^e^ 50 mM | 6.5 | 70 | 37-43 |
|  |  |  |  |  |  |  |
| ZgLam16A | 25, 36 | 5, 10 µg | Glycine-NaOH 100 mM | 8.5 | 40 | 15-20 |
|  | 36 | 10 µg | Sodium phosphate 50 mM | 6.5 | 40, 50 | 24-29 |

a. One unit of E-LAMHV activity is defined as the amount of enzyme required to release one µmole of glucose-reducing sugar equivalents per minute from laminarin β(1,3)-D-glucan (10 mg/mL) as substrate in 100 mM sodium acetate buffer, pH 5.0 at 40^o^C. The amounts of 50 and 100 U of E-LAMHV in 50 mM sodium acetate buffer, pH 5.0, 36 h at 37^o^C were also tested in the presence of 10, 20, 30 and 50% glycerol.

b. One unit of E-LICACT activity is defined as the amount of enzyme required to release one µmole of glucose-reducing sugar equivalents per minute from barley β-D-glucan (5 mg/mL) as substrate in 100 mM sodium phosphate buffer, pH 6.5 at 40*^o^C*.

c. MES: 2-(N-morpholino)ethanesulfonic acid.

d. CtLam81A 75 µg, in 50 mM phosphate buffer, pH 6.0, 36 h at 60^o^C was also tested in the presence of 10, 20 and 30% glycerol.

e. Tris-HCl: 2-amino-2-(hydroxymethyl)propane-1,3-diol hydrochloride.
